# High quality palliative alternative for patients with advanced locoregional (CN3 Stage) penile cancer

**DOI:** 10.1590/S1677-5538.IBJU.2021.0169.1

**Published:** 2021-10-01

**Authors:** Stênio de C. Zequi

**Affiliations:** 1 Fundação Prudente A. C. Camargo Cancer Center São PauloSP Brasil A. C. Camargo Cancer Center, Fundação Prudente, São Paulo, SP, Brasil

## COMMENT

In the article intitled “The role of inguinal surgical debulking for locally advanced penile cancer followed by reconstruction with miocutaneous flap” (1), authors must be congratulated by their efforts in favor of offering a high quality palliative alternative for patients with advanced locoregional (cN3 stage) penile cancer which are not able to seek or receive the medical attention as preconized by the most important urooncologic guidelines (neoadjuvant chemotherapy followed by extended inguinal lymphadenectomy) (2-4). Using the upfront radical surgical resection plus miocutaneous flap rotation, they avoid or postponed immediate local progression, reducing pain, infection and perhaps delaying the suffered death by difficult managed local complications.

However, this exclusive challenging surgical procedure is not enough to proportionate cure or an adequate locoregional control of disease. In this scenario of penile cancer, the multidisciplinary approach is unquestionably mandatory, as authors findings, that shown better overall survival and disease free survival (14 versus 6 months (p=0.0006), and 10 versus 3 months (p-0.002), respectively), for patients that received adjuvant chemotherapy.

Although this strategy seems reasonable for underdeveloped and for many developing countries, were this malignance is prevalent and systemic treatment usually is not disponible, it is far from desirable: for this approach is necessary to count with skilled surgical teams (urologic an plastic surgeons), intensive and nutritional care, for patients demanding prolongated hospital stay due their poor performance/status associated with the high rates of complication, which are inherent this kind of procedures. Additionally, concomitant results are expensive hospital expenses, and short disease-free survival and minimal overall survival.

Conversely the preconized neoadjuvant chemotherapy (5) has not been capable to offer long term results, sometimes the following surgery are so complex also, and in case of failure, the salvage therapeutics are ineffective or have short duration (6).

In the next years, more robust data are waited from the Eastern Cooperative Oncology Group (ECOG) trial EA8134 NCT02305654 (International Penile Cancer Adjuvant Chemotherapy Trial, the “InPACT” Trial), a large multinational Phase III study evaluating the roles of neoadjuvant chemotherapy or neoadjuvant chemoirradiation followed by surgery, versus upfront inguinal lymphadenectomy. And after inguinal treatment, it will be evaluated the roles of adjuvance with chemotherapy, radiotherapy, chemoirradiation, pelvic lymphadenectomy, or surveillance. We do not know if toxicities may be significant with theses associations of classic morbid and efficacy-limited traditional therapies.

An open question for the study from Rio de Janeiro group, what would the contribution from the probably pelvic metastases of their patients for the disease progression or cancer deaths described? How to treat these pelvic metastases considered uncurable by authors?

In this way, it seems more than necessary the emergency of new personalized therapies for penile cancer, as target therapies (anti-EGFR), immunotherapy (for PDL-1 positives or high mutational burden cases, e.g.) or new biomarkers, molecular signatures, that could be used in advanced cases (7-9).

Unfortunately, this future seems so far, in face of the high costs of these drugs; the low economical support for studies for these kinds of malignancies; the biological aggressiveness of this neoplasm; the difficult to implementation of clinical trials in some countries with large incidence of penile cancer, in which prevention is a hard task, yet.

## References

[1] 1. Koifman L, Hampl D, Ginsberg M, Castro RB, Koifman N, Ornellas P, et al. The role of primary inguinal surgical debulking for locally advanced penile cancer followed by reconstruction with myocutaneous flap. Int Braz J Urol. 2021;47; 1162-75. 10.1590/S1677-5538.IBJU.2021.0169 PMC8486458 34115458

[2] 2. Hakenberg OW, Compérat EM, Minhas S, Necchi A, Protzel C, Watkin N. EAU guidelines on penile cancer: 2014 update. Eur Urol. 2015;67:142-50. 10.1016/j.eururo.2014.10.017 25457021

[3] 3. NCCN Clinical Practice Guidelines in Oncology: Penile Cancer. 2019. V2. [Internet]. Available at. <https://www.nccn.org/professionals/physician_gls/pdf/penile.pdf>

[4] 4. Azevedo RA, Roxo AC, Alvares SHB, Baptista DP, Favorito LA. Use of flaps in inguinal lymphadenectomy in metastatic penile cancer. Int Braz J Urol. 2021;47; 1108-19. 10.1590/S1677-5538.IBJU.2021.99.14 PMC8486440 34115457

[5] 5. Pagliaro LC, Williams DL, Daliani D, Williams MB, Osai W, Kincaid M, et al. Neoadjuvant paclitaxel, ifosfamide, and cisplatin chemotherapy for metastatic penile cancer: a phase II study. J Clin Oncol. 2010;28:3851-7. 10.1200/JCO.2010.29.5477 PMC2940402 20625118

[6] 6. Wang J, Pettaway CA, Pagliaro LC. Treatment for Metastatic Penile Cancer After First-line Chemotherapy Failure: Analysis of Response and Survival Outcomes. Urology. 2015;85:1104-10. 10.1016/j.urology.2014.12.049 25819619

[7] 7. Gupta S, Sonpavde G. Emerging Systemic Therapies for the Management of Penile Cancer. Urol Clin North Am. 2016;43:481-91. 10.1016/j.ucl.2016.06.009 27717434

[8] 8. Ahmed ME, Falasiri S, Hajiran A, Chahoud J, Spiess PE. The Immune Microenvironment in Penile Cancer and Rationale for Immunotherapy. J Clin Med. 2020;9:3334. 10.3390/jcm9103334 PMC7603091 33080912

[9] 9. Chahoud J, Pickering CR, Pettaway CA. Genetics and penile cancer: recent developments and implications. Curr Opin Urol. 2019;29:364-70. 10.1097/MOU.0000000000000640 31045928

